# Simultaneous Tumor and Bilateral Medial Thalamic Radiosurgery for Tumor-Trigeminal Neuralgia With Concomitant Continuous Pain: A Case Report

**DOI:** 10.7759/cureus.110929

**Published:** 2026-06-15

**Authors:** Jesus Antonio Rodriguez Santos, Paola Del Cid, Alejandra Moreira, Victor Caceros, Jose Chang, Eduardo E Lovo

**Affiliations:** 1 Radiosurgery, International Cancer Center, San Salvador, SLV; 2 Neurosurgery, Pontificia Universidad Católica de Chile, Santiago, CHL; 3 Neurosurgery, Salvadoran Institute of Social Security, San Salvador, SLV

**Keywords:** concomitant continuous pain, gamma knife radiosurgery, medial thalamic radiosurgery, meningioma, radioneuromodulation, trigeminal neuralgia

## Abstract

Tumor-related trigeminal neuralgia (TN) may present with concomitant continuous pain (CCP), a persistent burning background pain between paroxysmal crises that can be particularly difficult to manage. When caused by skull base tumors involving the cavernous sinus and posterior fossa, complete encasement of the trigeminal nerve markedly increases therapeutic complexity, often rendering conventional options, such as microsurgical decompression, nerve-targeted radiosurgery, or percutaneous procedures, impractical or unsafe. In such settings, radiosurgical management may offer an alternative strategy capable of addressing both tumor control and pain relief. We report the case of a 41-year-old woman with severe refractory secondary TN and CCP caused by a right cavernous sinus meningioma extending into the temporal, infratemporal, and posterior fossae, with complete encasement of the trigeminal nerve along its entire intracranial course. Treatment consisted of simultaneous irradiation of the tumor and bilateral medial thalamic structures, delivering a maximum dose (Dmax) of 125 Gy to both thalami and a prescription dose of 14 Gy to the meningioma. Symptomatic improvement began within 35 hours of treatment, with complete pain relief (Visual Analog Scale (VAS) 0) achieved by day 6 and sustained throughout 39 months of follow-up. This case suggests that simultaneous tumor radiosurgery and bilateral medial thalamic irradiation may represent a feasible rescue strategy in selected patients with anatomically inaccessible tumor-related TN with CCP. Further study is needed to define the role of focal thalamic irradiation in the acute and long-term control of this challenging pain phenotype.

## Introduction

Concomitant continuous pain (CCP) is a relatively infrequent presentation of trigeminal neuralgia (TN) in which patients experience a persistent, debilitating background ache between paroxysms, a finding that may occur regardless of TN etiology and that has been associated with poor response to treatment [1]. Approximately 1%-9% of TN cases are caused by intracranial tumors, most commonly cerebellopontine angle (CPA) lesions [2]. Less commonly, a tumor may extend from the cavernous sinus through the middle and into the posterior fossa, completely encasing the trigeminal nerve and rendering it undetectable along its intracranial course. Although surgical resection remains the standard treatment for tumor-related TN and for symptomatic meningiomas, complete excision of skull base lesions is frequently unattainable and may carry substantial risk of cranial nerve morbidity without guaranteed pain relief. These limitations have driven growing interest in radiosurgical management for lesions in which resection poses unacceptable risk [3].

Gamma Knife radiosurgery (GKRS) has been established as an effective option for meningiomas, particularly skull base tumors, achieving five-year tumor control rates as high as 90.2% and improvement of pre-existing cranial neuropathy in 45.1% of cavernous sinus cases [3]. Because a subset of CPA tumors causes TN, GKRS has also become a primary treatment for intractable tumor-related TN in patients unwilling or unable to undergo surgery, providing pain relief within the first year with acceptable long-term efficacy. However, its effect typically requires a latency of several months, and persistent pain has been reported in approximately 20% of cases [2].

Prior reports have described unusually rapid responses to radiosurgery in a subset of TN patients, suggesting that focal radiation may acutely modulate neuronal function before classical radiobiological mechanisms can account for cellular death, whether by irradiating the nerve itself or the medial structures of the thalamus [4,5]. This phenomenon, which our group has termed radioneuromodulation, refers to the focal irradiation of pain-related circuitry to alter the function of living neurons rather than to ablate tissue, with the goal of rapid (50%) pain relief; it remains a proposed mechanism rather than an established one. The medial thalamic nuclei, including the centromedian-parafascicular complex (CM-Pfc) and adjacent mediodorsal structures, participate in the affective-motivational and modulatory processing of orofacial nociception through dense projections to limbic and prefrontal cortices, providing an anatomical rationale for their use as a radiosurgical target in refractory facial pain [5]. Different radiosurgical strategies have been described for tumor-related TN, including irradiation of the tumor alone or of the tumor and the nerve at different doses, with the tumor typically receiving a standard dose of 12-16 Gy and the nerve a high pain-control dose of 60-80 Gy [6,7]. In the case reported here, the principal challenge was refractory CCP combined with complete tumoral encasement of the entire intracranial trigeminal nerve, precluding any direct nerve-targeted intervention. Given the bilateral representation of the trigeminothalamic projections in the medial thalamic structures and our previous experience with unilateral irradiation of this region [5], we elected to perform bilateral medial thalamic irradiation simultaneously with tumor radiosurgery, aiming to achieve rapid pain relief together with oncologic control. To our knowledge, this is the first report of single-session simultaneous tumor and bilateral medial thalamic radiosurgery in a patient with tumor-related TN with CCP in whom the trigeminal nerve was radiographically inaccessible along its entire intracranial course; we present this case to illustrate the rationale, feasibility, and dosimetric considerations of this combined approach.

## Case presentation

A 41-year-old woman with no significant past medical history was referred from an outside institution. She presented with a three-month history of paresthesia involving the jaw and neck areas, triggered by teeth brushing, which progressively increased in duration and frequency until she sought dental evaluation in August 2021. Two weeks later, episodes were accompanied by sudden bouts of intense facial pain and swelling, categorized as an autonomic response, with a Visual Analog Scale (VAS) score of 10/10, predominantly affecting the right side of the face and scalp. Episodes lasted approximately 20 minutes and prevented her from continuing daily activities. A persistent dull, moderate ache (VAS 4-5/10) developed between episodes. She also developed diplopia, blurred vision, and reduced visual acuity, along with severe pain involving the right eye and the right side of the mouth and tongue, neck stiffness and swelling, hypersalivation, right-sided dysgeusia, painful mastication, and impaired voice modulation. 

She subsequently consulted a neuro-ophthalmologist and underwent her first magnetic resonance imaging (MRI) study (Figure 1), which demonstrated an extra-axial lesion with intense, homogeneous contrast enhancement located in the right cavernous sinus, extending into the temporal fossa, Meckel’s cave, and posterior fossa. The imaging features were most consistent with meningioma based on the extra-axial location, homogeneous enhancement, and dural-based growth pattern; schwannoma and hemangiopericytoma were considered less likely. Histopathological confirmation was not obtained because surgical resection was not undertaken. She was referred to a neurosurgeon and started on carbamazepine 100 mg/day for the first 10 days, then 200 mg/day, together with pregabalin 150 mg/day, meloxicam 15 mg/day, additional non-steroidal anti-inflammatory drugs, and occasional opioids such as oxycodone for breakthrough exacerbations. After two months of medical management, she experienced a particularly severe episode (VAS 10/10) that caused rigidity of the right scapula and arm and limited her ability to stand. She was then evaluated by a neuro-anesthesiologist; physical examination revealed an absent corneal reflex on the right as well as right-sided anacusis. A peripheral trigeminal nerve block was performed, which reduced the frequency of pain episodes and provided up to 5 pain-free days; however, blocks were required every 15 days, and dexamethasone was prescribed every 3 days.

**Figure 1:**
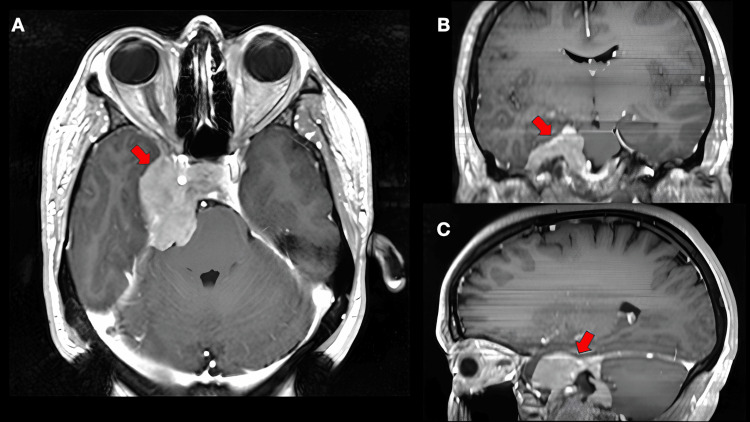
Preoperative gadolinium-enhanced MRI demonstrating a giant right cavernous sinus meningioma with multicompartmental extension. T1-weighted gadolinium-enhanced brain MRI in axial (A), coronal (B), and sagittal (C) planes. Images show an extra-axial, homogeneously contrast-enhancing tumor centered in the right cavernous sinus, with extension into the temporal and infratemporal fossae, Meckel’s cave, and posterior fossa, and complete encasement of the trigeminal nerve along its intracranial course.

In October 2021, the patient was referred to our center for neurosurgical evaluation. At that time, she presented with CCP secondary to right TN with parasympathetic manifestations and right sixth cranial nerve (CN VI) paresis. After multidisciplinary discussion with skull base and tumor neurosurgeons, no surgical approach was deemed feasible without unacceptable risk of new neurological deficit or incomplete pain relief; an alternative strategy of radiosurgical management was therefore considered. Treatment was ultimately scheduled in January 2023 following completion of the cross-border referral process, coordination of treatment logistics, and patient travel arrangements. In summary, the defining diagnostic features of this presentation were: refractory right-sided TN with CCP; progressive multiple cranial neuropathies (absent right corneal reflex, right anacusis, CN VI paresis) with parasympathetic manifestations; and MRI demonstrating a giant right cavernous sinus meningioma with multicompartmental skull base extension and complete intracranial encasement of the trigeminal nerve.

The patient underwent radiosurgical treatment with the Infini Rotating Gamma Ray Unit (Masep Medical Science & Technology Development Co., Shenzhen, China). The meningioma was treated with a prescription dose of 14 Gy at the 50% isodose line, achieving 95% target coverage with a conformality index of 0.860. Bilateral medial thalamic irradiation was performed using a single 4-mm collimator shot per side, prescribing a maximum dose (Dmax) of 125 Gy to each thalamus, corresponding to a biologically effective dose (BED) of 90.1 Gy at the time of treatment, considering cobalt decay. Thalamic targeting was based on indirect coordinates referenced to the anterior commissure-posterior commissure (AC-PC) line: 4 mm anterior to the PC on the Y-axis, 4.5-5 mm lateral to the ventricular wall on the X-axis, and 4 mm superior to the AC-PC plane on the Z-axis. Treatment planning was performed on the SuperPlan system (Masep Medical Science & Technology Development Co., Shenzhen, China) using gadolinium-enhanced T1-weighted MRI for target definition. Figure 1 demonstrates the preoperative radiological appearance of the lesion, highlighting its origin within the right cavernous sinus and its extensive skull base involvement. Figure 2 demonstrates the evolution from conventional atlas-based targeting to a connectomic-guided bilateral medial thalamotomy radiosurgical paradigm with improved dosimetric optimization. The illustrated treatment plans were not used in the management of the current patient and are included solely for comparative evaluation of dose distributions achieved with atlas-based versus actual connectomic-guided targeting approaches. A comparative dose-volume analysis between a reconstructed 125-Gy atlas-based bilateral medial thalamotomy plan and a contemporary 90-Gy connectomic-guided approach targeting the CM-Pfc is summarized in Table 1. The analysis includes dosimetric parameters for the bilateral periventricular gray nucleus (PVG-N) and ventral posterolateral/ventral posteromedial nucleus and is presented for methodological comparison only, as neither plan corresponds to the treatment delivered in the current case.

**Figure 2:**
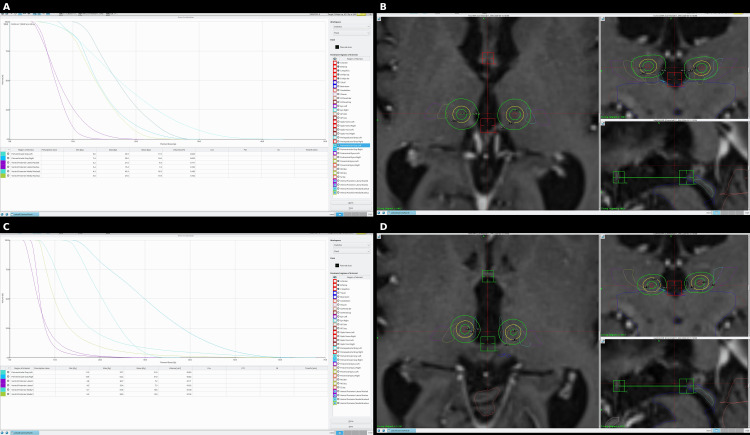
Evolution of bilateral medial thalamotomy radiosurgical planning from atlas-based targeting to connectomic-guided optimization. (A) Dose-volume histogram (DVH) illustrating a reconstructed atlas-based bilateral medial thalamic radiosurgical approach used in 2023 for comparison purposes. (B) Corresponding treatment plan demonstrating the conventional bilateral medial thalamic targeting strategy. (C) DVH of the contemporary connectomic-guided bilateral medial thalamic radiosurgery approach using a reduced prescription dose of 90 Gy. (D) Updated treatment plan illustrating the current connectomic-based targeting paradigm, reflecting improved dosimetric optimization and target refinement. These plans are presented solely for the methodological comparison of atlas-based versus connectomic-guided dose distributions and do not correspond to the radiosurgical treatment delivered to the patient described in this report.

**Table 1:** Comparative dose-volume analysis of two bilateral medial thalamic radiosurgical planning strategies: 125-Gy atlas-based reconstructed patient plan and a 90 Gy connectomic-guided approach directed at the centromedian-parafascicular complex. Dose-volume parameters are reported for the bilateral periventricular gray and the ventral posterolateral and ventral posteromedial nuclei. Neither plan corresponds to the radiosurgical treatment received by the patient described in this report; the analysis is intended solely to contrast the dose distribution achievable with atlas-based versus connectomic-guided targeting paradigms. Max: maximum dose, Mean: mean dose, Min: minimum dose.

Region of interest	125-Gy atlas-based reconstructed patient plan				90 Gy connectomic-guided plan (current practice)			
	Min (Gy)	Max (Gy)	Mean (Gy)	Volume (cm³)	Min (Gy)	Max (Gy)	Mean (Gy)	Volume (cm³)
Periventricular gray, left	9.6	29.3	17.5	0.023	11.3	37.7	21	0.023
Periventricular gray, right	7.4	30.2	14.6	0.022	13.4	62.2	31	0.022
Ventral posterolateral nucleus, left	3.2	21	8.3	0.171	2.8	30.7	7.2	0.171
Ventral posterolateral nucleus, right	3.4	15.4	7.4	0.162	4.3	32.4	7.3	0.162
Ventral posteromedial nucleus, left	4.3	43.3	18.3	0.1	6.1	67.8	16	0.1
Ventral posteromedial nucleus, right	3.8	27.6	13.9	0.102	5.2	59.5	12	0.102

## Discussion

Therapeutic challenges in tumor-related TN with CCP

TN with CCP represents a particularly challenging clinical phenotype because conventional therapies are less predictable and less durable than in purely paroxysmal disease. The presence of CCP has been associated with trigeminal nerve atrophy, sensory abnormalities, and a biology that appears to extend beyond ephaptic irritation alone, suggesting a more structurally and centrally dysregulated pain state [1]. In secondary TN, the presence of CCP may reflect a more complex pathophysiology beyond paroxysmal ectopic discharges, involving mechanisms of persistent neuropathic pain. A subset of TN cases arises from CPA tumors, whose deep location within the posterior fossa and close relationship to the brainstem and adjacent cranial nerves can make complete (Simpson grade I) resection particularly challenging, especially when extension into the cavernous sinus, internal auditory canal, or jugular foramen is present [3].

Why standard therapeutic routes failed in this case

In this patient, the usual therapeutic hierarchy in TN had, in effect, been anatomically eliminated. Surgical decompression could not be achieved without unacceptable risk because the tumor extended across the anterior, middle, and posterior skull base, with no corridor offering a complete solution: a posterior approach would have left the nerve compromised within Meckel’s cave and the cavernous sinus, while an anterior or middle fossa route would have exposed the patient to a substantial risk of new neurological deficit. Radiosurgery to the trigeminal nerve itself was not feasible because the nerve was not identifiable in any of its intracranial portions and therefore could not be safely targeted. Percutaneous rhizotomy was also not feasible because Meckel’s cave was completely occupied by the tumor, precluding safe anatomical access for the procedure. Given these constraints, our multidisciplinary team considered simultaneous radiosurgery targeting both pain control and tumor treatment to be the most efficient strategy. Our prior experience with medial thalamic irradiation for refractory TN [5] supported bilateral targeting in this case, given the bilateral representation of orofacial nociceptive input within the medial thalamus. 

Rationale for radioneuromodulation

The observation that trigeminal pain control is often best immediately after radiosurgical management appears to contradict the known mechanisms of radiosurgical effects on tissue, as radiobiological lesion creation and effects on synaptic activity are typically slower. This has led to the proposal that radiation may exert a neuromodulatory effect on certain neurons. Radioneuromodulation, as defined by our group, is a pain management technique based on focal irradiation of specific pain circuitry to alter the function of living neurons and glia, aiming to provide rapid (<72 hours), substantial (>50%) pain relief. Animal studies have shown that radiomodulatory doses can shorten action potentials, inhibit sodium channel activity, and reduce synaptic transmission, effects that may be relevant to the management of CCP in TN [8].

Our center has previously achieved radioneuromodulation in TN patients in whom nerve-targeted radiosurgery had failed, as well as in those for whom tumor involvement or prior nerve sectioning ruled out surgical or percutaneous approaches [5]. Delivery of high doses (90 Gy) to the affected nerve combined with 120-140 Gy to the contralateral medial thalamic structures produced rapid pain relief and improved quality of life in 100% of patients in a small series with CCP [9,10]. This dual-target approach was informed by prior experience with single-target strategies for refractory TN with CCP, which were often associated with delayed clinical improvement, as well as by previous results with isolated thalamic irradiation in classic TN [5]. The distinguishing feature of the present case is the use of bilateral medial thalamic irradiation in the setting of complete tumoral encasement of the trigeminal nerve. In our prior experience, unilateral thalamic irradiation produced inconsistent and often transient radiomodulatory effects, with definitive pain relief typically emerging approximately three months after treatment [5]. Radiosurgery targeting both the trigeminal nerve and thalamus was not feasible because no identifiable trigeminal nerve segment was visible on high-resolution MRI. Tumor-only irradiation was also unlikely to provide sufficiently rapid pain relief, particularly given the patient’s planned return to her country of origin, where close follow-up and salvage treatment could not be ensured. 

Outcomes of lone tumor radiosurgery

Although radiosurgery is well established for tumor control, its effect on tumor-related TN is variable. Following tumor-only irradiation, reported pain relief rates range from 33% to 96%, and benign tumors respond more slowly, with mean latencies of 3-5 months before symptomatic relief; the behavior of CCP crises after lone tumor management has not been well characterized [6]. Reductions in tumor volume have not shown a reliable association with pain control, and relief often precedes any significant shrinkage, implying that targeting the tumor alone may be insufficient for durable pain control in tumor-related TN [6]. Repeat radiosurgery targeting the trigeminal nerve has been recommended in such cases [7]. Kim et al. improved the durability of pain relief without increasing toxicity by targeting both the trigeminal nerve and the tumor in a single session, reducing pain recurrence at three years to 21.4% [7]. 

The medial thalamus as a target for refractory pain

The medial thalamic structures modulate the cognitive and emotional processing of orofacial pain through projections to limbic regions, including the prefrontal cortex, temporal cortex, insula, parahippocampal gyrus, and anterior cingulate cortex [11,12]. In TN patients, significant gray-matter reduction in these limbic regions, including the amygdala, has been observed and suggests pathological impairment of descending pain modulation, leading to heightened pain and emotional responses. This dysfunction has been implicated in the transition from acute pain to chronicity, consistent with a gradual shift from nociceptive to affect-related circuitry in chronic pain states [11].

Prior reports have suggested medial thalamic radiosurgery as a promising and safe option for neuropathic pain, with the most favorable outcomes reported in trigeminal deafferentation pain [13]. Our group has found that irradiation of the mediodorsal nucleus (MD), CM-Pfc region with 140-150 Gy provides substantial pain improvement in 60% of patients within days of treatment, a response too rapid to be explained by classical radiation-induced damage, supporting a neuromodulatory mechanism [5]. Most of these patients subsequently experienced trigeminal pain crises or pain recurrence over the ensuing months, with the more stable radiosurgical effect typically appearing at approximately three months [5]. We have additionally used isodose-curve analysis to identify probable areas of therapeutic influence, placing the 70 Gy (50% isodose) line within the CM-Pfc region most closely interconnected with the MD and the ventroposteromedial nucleus (VPM), so that after adjustment for dose gradients, the 20 Gy isodose line still encompasses additional thalamic regions potentially relevant to pain regulation, including the periventricular gray nucleus (PVG-N) [14]. While the minimum radiation dose required to induce a radiomodulatory effect remains undefined, experimental data show that sub-ablative focal irradiation can alter neural circuit function without overt tissue necrosis [15].

Evolution of dose strategy and individualized targeting

Earlier radiosurgical pain models used relatively high thalamic doses based on the assumption that a meaningful biological effect required doses approaching lesioning thresholds [16]. Experimental and clinical reasoning supported the view that doses above 60 Gy could alter neuronal activity, while doses above 100 Gy tended to evolve toward tissue necrosis over time [8,16]. Based on our prior institutional experience, this led us to favor doses above 120 Gy for CCP-predominant trigeminal pain [5,9]. More recent connectomic-guided work has refined this framework. Rather than assuming that the maximal biological effect depends primarily on a high central dose, individualized planning suggests that clinical response may depend equally on which connected structures fall within the surrounding area of influence, particularly at lower isodose volumes such as the 20 Gy region encompassing pain-relevant nodes, including the VPM, ventral posterolateral nucleus (VPL), and PVG-N. Effective radiosurgical neuromodulation may therefore not always require maximal ablative doses, but rather a precise match between dose distribution and patient-specific pain circuitry [14].

A major methodological shift occurred with the incorporation of Brainlab Elements (Munich, Germany) autosegmentation tools for the thalamus, which for the first time enabled systematic dose-volume histogram (DVH) analysis of some of the individual nuclei involved in nociceptive processing. This capability allowed us to quantify the dose delivered to the descending pain pathway through the periventricular gray, as well as to the lateral sensory pathways through the VPM and the VPL. Although Brainlab Elements does not currently support autosegmentation of the medial thalamic structures such as the CM-PFc, which precludes direct dosimetric measurement of the medial pathway, the available DVH data nonetheless provide, for the first time, an objective approximation of the dose actually deposited along the pain-relevant circuitry [14].

This evolution is illustrated in Figure 2 and was previously mentioned at the Case Presentation, which contrasts the bilateral medial thalamic strategy used in the present case in 2023 with our current connectomic-guided approach through their DVH and corresponding treatment plans. The historical plan relied on atlas-based stereotactic coordinates and effectively targeted the area MD of the thalamus, whereas the contemporary approach uses individualized connectivity and patient-specific thalamic anatomy to direct the dose predominantly at the CM-Pfc region [14]. The dosimetric analysis shown in Figure 2 was performed in Leksell GammaPlan (Elekta, Stockholm, Sweden) as a comparative exercise; it does not pertain to the actual clinical management of this patient, but rather serves to illustrate why our current practice favors lower prescription doses [14]. Despite a lower prescribed dose (90 Gy vs 125 Gy), the connectomic-guided plan delivered higher maximum doses across all evaluated pain-network structures and generally higher minimum doses in most regions (Table 1). Overall, this translated into approximately 20% greater dose delivery to the segmented pain-relevant nuclei compared with the old atlas-based plan. This redistribution illustrates how anatomically and functionally guided targeting can concentrate dose on the structures most relevant to neuromodulation while reducing the central peak dose [14].

The 125 Gy Dmax bilaterally prescribed in this patient reflected the conceptual framework and technological resources available at the time and was consistent with our institutional experience for refractory CCP. The rapid, profound, and durable pain relief achieved without observed toxicity suggests that this strategy was successful in this individual case. Current connectomic evidence nonetheless suggests that future similar cases may benefit from more individualized targeting and dose-volume optimization rather than dose escalation alone [14]. Whether bilateral treatment is superior to unilateral treatment remains uncertain. However, the bilateral cortical representation of orofacial pain and potential ipsilateral trigeminothalamic projections provide an anatomical rationale for bilateral modulation in selected cases [17].

Dose distributions encompassing the proposed areas of neuromodulation in a concentric isodose configuration may also affect both paleospinothalamic and spinothalamocortical pathways, potentially down-regulating hypermetabolic thalamocortical connectivity and thereby addressing both the affective and primary sensory components of facial pain. Given the aberrant connectivity reported in chronic pain states between mediodorsal thalamic structures and cortical areas such as the medial prefrontal cortex, anterior cingulate cortex, and insula, modulation of these networks may be particularly relevant for the relief of CCP-predominant TN [12].

Within this framework, our subsequent experience with bilateral medial thalamotomy and anterior cingulotomy for refractory pain has informed gradual refinement of our dose practice toward more individualized regimens [18]. The critical determinant of effect may lie less in producing a lesion and more in accurately engaging the relevant pain network. Similarly, our current approach to a tumor of this size would favor a staged or fractionated regimen to improve the therapeutic ratio [19]; however, at the time of this patient's treatment, fractionation was not technically available, and the delivered strategy reflected the resources of that moment. With respect to safety, we have not observed complications when targeting the medial thalamic region either unilaterally or bilaterally, consistent with the favorable safety profile reported in modern Gamma Knife thalamotomy series [20]. While the long-term durability of pain relief remains difficult to predict, recurrence rates reported by other groups [13] suggest that even an imperfectly durable response may represent a meaningful benefit in patients with intractable pain, particularly when conventional surgical, radiosurgical, and percutaneous routes have all been lost.

Limitations

This report has several important limitations. First, as a single-patient observation, the findings cannot be generalized and require corroboration in larger series. Second, although the rapid pain response is more biologically consistent with thalamic neuromodulation than with a direct tumor effect, a contributory effect from tumor irradiation alone cannot be excluded. An alternative staged approach, consisting of initial tumor irradiation followed by medial thalamic radiosurgery 3-6 months later if pain persisted, might have helped distinguish these effects. This strategy was not pursued because of the severity of the patient’s pain, travel limitations, and financial considerations. Third, radiological follow-up has been limited: although the patient was recently reassessed clinically and reported sustained pain resolution, long-term tumor control and volumetric response remain undetermined, as no recent follow-up brain MRI has been available for objective evaluation. Fourth, the diagnosis of meningioma rests on imaging characteristics rather than histopathology.

## Conclusions

This report describes an anatomically exceptional case of secondary TN with CCP in which complete tumoral encasement of the nerve effectively eliminated the standard therapeutic routes of management. Microsurgical decompression or nerve liberation carried an unacceptably high risk of morbidity, radiosurgical targeting of the trigeminal nerve was not feasible because the nerve could not be identified, and percutaneous rhizotomy was rendered impractical by complete tumoral compromise of Meckel’s cave. In that context, simultaneous radiosurgical treatment of the meningioma and bilateral medial thalamic irradiation provided rapid and durable pain relief together with sustained clinical stability of the tumor. Because recent volumetric imaging was not available, radiological tumor control could not be objectively confirmed and is inferred from the patient's sustained clinical course rather than demonstrated. Illustrating that in selected patients with refractory tumor-related trigeminal neuralgia, especially when the nerve is anatomically inaccessible, radiosurgery may function not only as a tumor treatment but also as a circuit-level pain strategy.

Although this single case cannot define a standard of care, it supports the idea that bilateral medial thalamic radiosurgery, performed simultaneously with tumor irradiation, may offer a meaningful rescue option for carefully selected patients in whom conventional surgical, radiosurgical, and percutaneous approaches to the trigeminal nerve have been effectively lost. Our current practice has since evolved toward more conservative bilateral thalamic dosing and a preference for staged or fractionated tumor treatment when technically feasible. Further study is needed to determine optimal target selection, dose architecture, and the long-term durability of pain control in this rare but highly challenging clinical scenario.
